# Psychological rumination and recovery from work in intensive care professionals: associations with stress, burnout, depression and health

**DOI:** 10.1186/s40560-017-0209-0

**Published:** 2017-02-02

**Authors:** Tushna Vandevala, Louisa Pavey, Olga Chelidoni, Nai-Feng Chang, Ben Creagh-Brown, Anna Cox

**Affiliations:** 1 School of Social and Behavioural Sciences, Criminology and Sociology, Faculty of Arts & Social Sciences, Kingston University, Penrhyn Road, Kingston, Surrey KT1 2EE UK; 2 School of Health Sciences, Faculty of Health and Medical Sciences, University of Surrey, Guildford, Surrey GU2 7XH UK; 3 Intensive Care Unit, Royal Surrey County Hospital, Egerton Road, Guildford, Surrey GU2 7XX UK; 4 Surrey Perioperative Anaesthesia Critical Care Collaborative Research Group (SPACeR), Department of Clinical and Experimental Medicine, Faculty of Health and Medical Sciences, University of Surrey, Guildford, GU2 7XH UK

**Keywords:** Intensive care, Critical care, Stress, Burnout, Health, Rumination

## Abstract

**Background:**

The work demands of critical care can be a major cause of stress in intensive care unit (ICU) professionals and lead to poor health outcomes. In the process of recovery from work, psychological rumination is considered to be an important mediating variable in the relationship between work demands and health outcomes. This study aimed to extend our knowledge of the process by which ICU stressors and differing rumination styles are associated with burnout, depression and risk of psychiatric morbidity among ICU professionals.

**Methods:**

Ninety-six healthcare professionals (58 doctors and 38 nurses) who work in ICUs in the UK completed a questionnaire on ICU-related stressors, burnout, work-related rumination, depression and risk of psychiatric morbidity.

**Results:**

Significant associations between ICU stressors, affective rumination, burnout, depression and risk of psychiatric morbidity were found. Longer working hours were also related to increased ICU stressors. Affective rumination (but not problem-solving pondering or distraction detachment) mediated the relationship between ICU stressors, burnout, depression and risk of psychiatric morbidity, such that increased ICU stressors, and greater affective rumination, were associated with greater burnout, depression and risk of psychiatric morbidity. No moderating effects were observed.

**Conclusions:**

Longer working hours were associated with increased ICU stressors, and increased ICU stressors conferred greater burnout, depression and risk of psychiatric morbidity via increased affective rumination. The importance of screening healthcare practitioners within intensive care for depression, burnout and psychiatric morbidity has been highlighted. Future research should evaluate psychological interventions which target rumination style and could be made available to those at highest risk. The efficacy and cost effectiveness of delivering these interventions should also be considered.

## Background

Majority of admissions into ICU are unplanned emergencies where ICU professionals are often required to rapidly attend to complex situations of uncertain outcomes. Several international studies have found that professionals working in ICU experience high levels of stress, moral distress, burnout, anxiety, depression and posttraumatic stress disorder [1–10]. Environmental factors (e.g. heavy workload, long working hours), patient factors (e.g. critical illness or end of life, witnessing pain and suffering from futile treatments) and ethical issues relating to communication lead to ICU professionals experiencing moral distress, burnout, ill health and staff turnover [2, 7, 11–15]. Uncertainty and responsibilities associated with end-of-life (EOL) decisions are associated with an increased burden [16]. A recent systematic review found that working in an intensive care setting correlated with substantial risks of emotional distress [8, 17].

Work recovery or unwinding from work is a process that facilitates psychological and physical restoration, and the impairment of recovery from work stress may result in poor health [18]. The recovery process is largely influenced by the extent to which individuals disengage (or disconnect) from their work demands and related thoughts [19]. Rumination can be defined as “passively and repetitively focusing on one’s symptoms of distress and the circumstances surrounding these symptoms” ([20], p. 855). Ruminative response to stress has been identified as contributing to the development and maintenance of depression [21], impaired somatic and mental health [22] and increase in work-related fatigue [23]. Evidence suggests the importance of rumination as a mediator [24, 25], while other studies have failed to find a mediation effect of work-related rumination [19]. Rumination per se may not be associated with impaired health, but the emotional component of rumination may evoke negative effects of other stressors [26].

Previous research has shown clear associations between stress, burnout and poor psychological health and has found these symptoms to be highly prevalent in ICU healthcare professionals. In this study, we aimed to confirm these associations and to investigate the potential mediating process of rumination style. We predicted that the association between work stressors, burnout, depression and risk of psychiatric morbidity would be mediated by rumination.

This study’s objectives were

1. To determine the association between ICU stressors, burnout, depression and risk of psychiatric morbidity
2. To determine the mediating effects of the three types of work-related rumination (affective rumination, problem-solving pondering and distraction detachment) on the relationship between ICU stressors and each of the outcome variables (burnout, depression and risk of psychiatric morbidity)
3. To determine the impact of occupational role (doctors vs. nurses), working hours (more than 40 h per week vs. 40 h per week or less) and gender (male vs. female) on rumination (affective rumination, problem-solving pondering, distraction detachment), ICU stressors, burnout, depression and risk of psychiatric morbidity

## Methods

### Design

A cross-sectional design was used.

### Participants

The sample consisted of 96 professionals working in ICU (46 males and 50 females; 58 doctors and 38 nurses) in three different hospitals in the UK. The majority of the doctors and nurses were aged between 31 and 50 years and married. The sample had a range of years of experience in intensive care ranging from 0–5 years to more than 20 years. Fifty-four participants (56.3%) worked a 40-h week or less, while 41 participants (42.7%) worked more than 40 h per week (one missing data for work hours). For full participant statistics, see Table 1.

**Table 1:** Participants demographic characteristics (*N* = 96)

Variable		Doctors	Nurses	Overall
Gender	Male	40 (69%)	6 (15.8%)	46 (47.9%)
	Female	18 (31%)	32 (84.2%)	50 (52.1%)
Age (years)	18–30	4 (6.9%)	13 (34.2%)	17 (17.7%)
	31–50	48 (82.8%)	24 (63.2%)	72 (75%)
	51–65	6 (10.3%)	1 (2.6%)	7 (7.3%)
Marital status	Single	4 (6.9%)	8 (21.1%)	12 (12.%)
	Married	44 (75.9%)	20 (52.6%)	64 (66.7%)
	In a relationship	9 (15.5%)	7 (18.4%)	16 (16.7%)
	Divorced	1 (1.7%)	3 (7.9%)	4 (4.2%)
Experience in ICU	0–5 years	15 (25.9%)	20 (52.6%)	35 (36.5%)
	6–10 years	17 (29.3%)	10 (26.3%)	27 (28.1%)
	11–20 years	15 (25.9%)	5 (13.2%)	20 (20.8%)
	>20 years	11 (19%)	3 (7.9%)	14 (14.6%)
Work hours	≤40 h/week	23 (39.7%)	31 (81.6%)	54 (56.3%)
	>40 h/week	34 (58.6%)	7 (18.4%)	41 (42.7%)

### Materials

The *General Health Questionnaire* (GHQ-12) developed by Goldberg was used to assess the risk of psychiatric morbidity. The GHQ-12 is a 12-item, self-administered questionnaire designed to detect risk for non-psychotic psychiatric morbidity in non-clinical adult populations [27]. It measures psychiatric symptoms such as depression, sleep disorders and loss of self-confidence. Responses were coded as 0 (e.g. better or same as usual) or 1 (e.g. less than usual, much less than usual) and summed to give an overall score ranging from 0 to 12, as recommended by the authors. In the present study, the tool demonstrated satisfactory reliability, Cronbach’s *α* = .81.

The *Oldenburg Burnout Inventory* (OLBI) developed by Demerouti et al. [28] is a well-validated psychometric tool that assesses burnout syndrome and contains two subscales of exhaustion and disengagement. Exhaustion subscale refers to emotions of emptiness, the need to take time off from work and physical symptoms of exhaustion. Disengagement subscale refers to negative and cynical views towards work [28]. The items for each subscale were summed to give an overall score ranging from 0 to 32, Cronbach’s *α* = .69 (disengagement) and *α* = .73 (emotional exhaustion).

The *Inventory of Depressive Symptomatology, Self-Reported* (IDS-SR) developed by Rush et al. is a 30-item self-reported scale which measures depressive signs and symptoms [29]. This inventory is useful in evaluating the severity of depression, and the cut-off score in detecting endogenous depression suggested by Rush et al. [30] is adopted in the specific study. Items were summed to give a total score ranging from 0 to 90, with satisfactory reliability, Cronbach’s *α* = .86.

The *ICU-related stressors questionnaire* developed by Coomber et al. [2] was used to identify the frequency and the stress severity of ICU-specific factors. Participants were asked to indicate how often they deal with (0 = never, 1 = occasionally, 2 = often) and how stressful they perceive (0 = not at all, 1 = slightly, 2 = moderately, 3 = very, 4 = extremely) 30 ICU-specific situations, such as bed allocation, dealing with death, treatment withdrawal and effects of stress/hours on personal/family life were included. An overall score was calculated by multiplying the frequency and stress ratings for each stressor and summing the totals, giving an ICU stressor score ranging from 0 to 240, Cronbach’s *α* = .84.

The *Work-Related Rumination Questionnaire* (WRRQ) developed by Cropley et al. [26] explores the unwinding process of switching off from work and evaluates one’s tendencies and directions to ruminative thinking. It consists of three subscales: (1) affective rumination refers to the state of fatigue and distress that participants experience when they think of issues related to work; (2) problem-solving pondering focuses on the logic and cognitive way of organising work issues; and (3) distraction detachments focuses on the unwinding process that takes place after the individual has left the work environment. Items were summed to give a total score of 24 for each subscale (affective rumination: Cronbach’s *α* = .83; problem-solving pondering: Cronbach’s *α* = .43; distraction detachment: Cronbach’s *α* = .76).

#### General personal information

Participants were asked general personal information such as age, gender, marital status, specialty and years of experience within ICU. Participants were asked how many hours per week they work at the indicated job (less than 40 per week, 40 per week, more than 40 per week) and how often do they face EOL decision-making procedures (once every week, twice a week, more than twice a week, once a day, more than once a day).

### Procedure

The permission of heads of the ICU departments in hospitals within four National Health Service (NHS) Trusts in the South of England was sought before an invitation letter was sent to their staff. The information sheet and invitation letter was sent to all prospective participants inviting them to complete an online questionnaire. A researcher also visited the hospitals to increase response rate and administered paper versions of the questionnaires. The online questionnaire was also made openly available to doctors who were registered with https://www.doctors.net.uk (an online database of over 220,000 doctors in the UK). Web-based questionnaires which are open to all users make calculation of a response rate more difficult, and in this instance, it was not possible. There were no differences in demographic characteristics between participants recruited online and in person. Ethical review and governance permissions were sought and received from the Faculty of Arts and Human Sciences, University of Surrey Ethics Committee (1003-PSY-14), the Faculty of Arts and Social Sciences, Kingston University Ethics Committee (1314/5/3) and the Research and Development Department of the participating NHS Trust in the South of England.

### Statistical analysis

Bivariate correlations were conducted to determine the associations between ICU stressors, burnout, depression and risk of psychiatric morbidity. Regression, mediation and moderation analyses were then conducted using the PROCESS software [31] to test the indirect effect of ICU stressors on each of the outcome variables via the types of work-related rumination. To determine the impact of occupational role, working hours and gender on rumination, ICU stressors, burnout, depression and risk for psychiatric morbidity, *t* tests were conducted with the Bonferroni correction for multiple comparisons.

## Results

### Sample characteristics

The means and standard deviations for each of the measured variables by occupational role, working hours and gender are shown in Table 2. After applying cut-off scores for risk of psychiatric morbidity and depression, 32.3% of the sample was at risk of psychiatric morbidity and 18.8% were at risk of depression.

**Table 2:** Differences in ICU stressors and burnout between gender, occupation type and working hours

	Overall mean (SD)	Doctors’ mean (SD)	Nurses’ mean (SD)	Males	Females	≤40 h per week	>40 h per week
ICU stressors	49.5 (26.09)	45.50 (24.63)	55.84 (27.43)	46.38 (28.37)	52.55 (23.67)	39.71 (15.69)	53.83 (27.64)
Burnout: emotional exhaustion	20.12 (3.09)	19.91 (3.14)	20.45 (3.04)	19.48 (3.49)	20.72 (2.57)	19.71 (3.01)	20.46 (3.35)
Burnout: disengagement	17.65 (3.1)	17.60 (3.01)	17.71 (3.28)	17.67 (2.95)	17.62 (3.26)	16.90 (2.76)	18.07 (3.42)

### The effects of ICU stressors on burnout, depression and risk of psychiatric morbidity

Correlation analyses indicated that ICU stressors were significantly associated with burnout (emotional exhaustion, *r*(96) = .43, *p* < .001 and disengagement, *r*(96) = .42, *p* < .001). Logistic regression analysis indicated that ICU stressors were a significant predictor of depression, *β* = .04, *p* = .002, but were not associated with the risk of psychiatric morbidity, *β* = .01, *p* = .353.

### The effects of gender, occupational role and working hours

Independent sample *t* tests were conducted to assess any differences in ICU stressors and burnout (emotional exhaustion and disengagement) between gender (males vs. females), occupation (doctors vs. nurses) and working hours (40 h per week or less vs. more than 40 h per week). There was a significant difference in burnout according to gender (emotional exhaustion), *t*(94) = −2.00, *p* = .049, with female ICU workers showing greater emotional exhaustion than male ICU workers. There were no other significant gender differences, and no significant differences between nurses and doctors for any of the other variables (the means and standard deviations are displayed in Table 2). There was a significant difference between those working 40 h per week or less and those working more than 40 h in ICU stressors, *t*(68) = −2.63, *p* = 0.011, with those working more than 40 h reporting greater ICU stressors than those working 40 h per week or less (means and standard deviations are displayed in Table 2). Part-time workers (*N* = 28) were excluded from this analysis due to the qualitatively different nature of the work and stress they encounter.

Chi square analyses were conducted to determine the differences in the risk of psychiatric morbidity and depression between gender (males vs. females), occupation (doctors vs. nurses) and working hours (40 h per week or less vs. more than 40 h per week). There was a significant association between gender and risk of psychiatric morbidity, with females displaying a greater incidence of being at risk of psychiatric morbidity than males, *Χ* ^2^(1) = 8.97, *p* = .003. There was also a significant association between occupational role and risk of psychiatric morbidity, with nurses displaying a greater incidence of being at risk of psychiatric morbidity than doctors, *Χ* ^2^(1) = 4.46, *p* = .035. There was no significant association between working hours and risk of psychiatric morbidity, and no significant associations between gender, occupation, working hours and depression.

### The mediating and moderating role of rumination styles

Four mediation analyses were conducted to determine the mediating effects of the work-related rumination (affective rumination, problem-solving pondering and distraction detachment) on the relationship between ICU stressors and each of the four outcome variables. The full correlation matrix is displayed in Table 3. Linear regression was used for the two continuous outcome variables (emotional exhaustion and disengagement, see Table 4), and logistic regression for the two binary variables (depression and risk of psychiatric morbidity, see Table 5).

**Table 3:** Bivariate correlations for continuous predictor, mediating and outcome variables (*N* = 96)

	2	3	4	5	6
1. ICU stressors	.47**	.17	.31**	.43**	.42**
2. Affective rumination	–	.45**	.50**	.58**	.40**
3. Problem-solving pondering		–	.35**	.21*	.02
4. Distraction detachment			–	.36**	.08
5. Emotional exhaustion				–	.62**
6. Disengagement					–

*p < .05; **p < .01

**Table 4:** Linear regression models examining the effect of ICU stressors on burnout (emotional exhaustion and disengagement), mediated by rumination style (affective rumination, problem-solving pondering and distraction detachment)

	Emotional exhaustion		Disengagement	
	*β*	*t*	*β*	*t*
Step 1				
ICU stressors	0.43	4.26**	0.42	4.16*
*R* ^2^	.43**		.42**	
Step 2				
ICU stressors	0.17	1.73	0.26	2.41*
Affective rumination	0.53	4.50**	0.45	3.49**
Problem-solving pondering	−0.05	−0.52	−0.13	−1.24
Distraction detachment	0.06	0.57	−0.09	−0.81
*R* ^2^	.64**		.54**	

*p < .05; **p < .01

**Table 5:** Logistic regression models examining the effect of ICU stressors on depression and risk of psychiatric morbidity, mediated by rumination style (affective rumination, problem-solving pondering and distraction detachment)

	Depression			Risk of psychiatric morbidity		
	*β*	Wald	Odds ratio	*β*	*t*	Odds ratio
Step 1						
ICU stressors	0.35	8.41**	1.04	0.01	0.86	1.00
Nagelkerke *R* ^2^	.17**			.02		
Step 2						
ICU stressors	0.02	2.40	1.02	−0.01	0.44	.99
Affective rumination	0.34	10.78**	1.40	0.21	5.60*	1.23
Problem-solving pondering	−0.14	0.09	.87	−0.03	0.13	.97
Distraction detachment	−0.09	0.09	.14	0.07	0.57	1.07
Nagelkerke *R* ^2^	.37**			.18*		

*p < .05; **p < .01

Results showed significant effects of ICU stressors on affective rumination (*β* = .47, *t* = 4.74, *p* < .001) and distraction detachment (*β* = .31, *t* = 2.85, *p* = .005), but not on problem-solving pondering (*β* = .17, *t* = 1.60, *p* = .113).

When ICU stressors and the three mediating variables were added to each regression model simultaneously, affective rumination was the only variable to significantly predict emotional exhaustion (*β* = .53, *t* = 4.50, *p* < .001), disengagement (*β* = .45, *t* = 3.49, *p* < .001), depression (*β* = .44, *z* = 3.14, *p* = .002) and risk of psychiatric morbidity (*β* = .21, *z* = 2.37, *p* = .018). The effect of ICU stressor on each of the outcome variables diminished when rumination styles were added to the analysis (see Tables 4 and 5).

**Fig. 1:**
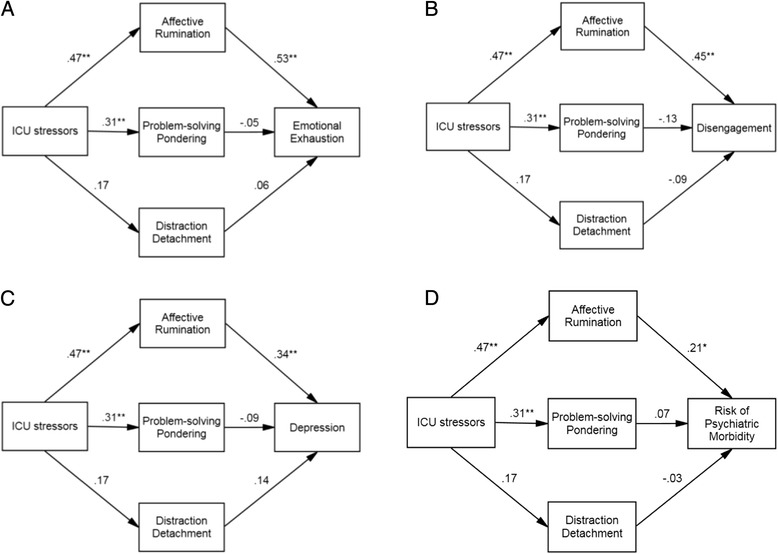
Path models with standardised beta weights showing the indirect effects of ICU stressors on **a** emotional exhaustion, **b** disengagement, **c** depression and **d** risk of psychiatric morbidity. *p < .01; **p < .001

Inspection of the indirect effects revealed a significant effect of ICU stressors on emotional exhaustion via affective rumination (95% CI [0.01, 0.06]) but not via problem-solving pondering (95% CI [−0.01, 0.01]) or distraction detachment (95% CI [−0.01, 0.01]). There was also a significant effect of ICU stressors on disengagement via affective rumination (CI 0.01, 0.06) but not via problem-solving pondering (CI −0.01, 0.001) or distraction detachment (CI −0.001, 0.01). The same result was found for depression: there was a significant indirect effect found between ICU stressors and depression via affective rumination (95% CI [0.01, 0.07]) but not via problem-solving pondering (95% CI [−0.01, 0.02]) or distraction detachment (95% CI [−0.03, 0.02]). Although there was no significant direct effect of ICU stressors on risk of psychiatric morbidity, there was a significant indirect effect of ICU stressors on risk of psychiatric morbidity via affective rumination (95% CI [0.01, 0.03]) but not via problem-solving pondering (95% CI [−0.01, 0.01]) or distraction detachment (95% CI [−0.01, 0.02]). Path models with standardised beta weights are shown in Fig. 1.

Overall, the results suggest that affective rumination, but not problem-solving pondering or distraction detachment, significantly mediated the effect of ICU stressor on each of the outcome variables (burnout, depression and risk of psychiatric morbidity). Moderation analysis revealed no significant moderation effects of rumination style on the association between ICU stressors and the four outcome variables.

## Discussion

The findings from the current study indicated that staff working in an ICU can experience significant stress and this was negatively associated with health and wellbeing: 32% of the sample were categorised as being at risk of psychiatric morbidity and 18% as being at risk of depression. These findings are comparable with empirical evidence that between 20 and 30% of ICU professionals are at risk of psychiatric morbidity [1, 2] and 10–25% express depressive symptoms [2, 32]. Prevalence estimates for common mental disorder from population studies range from 14 to 31%, with the prevalence of psychiatric morbidity in health professionals approximately 30% [33]. Participants who worked more than 40 h per week reported experiencing higher levels of ICU stressors, and ICU stressors were associated with higher incidences of burnout and depression. The current study did not account for shift patterns and night working but showed evidence that working in excess of 40 h may leave little time for actual recovery from work. It is possible that those working weekends or shift pattern may experience increased fatigue, irritability, decreased work efficiency and reduced mental agility [34].

The mediation analysis from the current study showed that ICU stressors per se may not lead to negative health outcomes and highlighted the importance of rumination style. Our findings corroborate and build on previous research suggesting that rumination is an important link between stress at work and negative health outcomes [24]. Our study reports that there were significant indirect effects of ICU stressors on burnout, depression and risk of psychiatric morbidity via affective rumination, but not via problem-solving pondering or distraction detachment. The results suggest that affective rumination style may hinder the process of recovery from work, leading to negative psychological health outcomes. Even though the maladaptive nature of rumination has been previously stressed, it remains a debate whether there are some adaptive aspects to it. Previous research has suggested that problem-solving pondering expresses a creative aspect of rumination that enables the individual to engage with the task and gain an enjoyable experience [19]. Unlike affective rumination, problem-solving pondering may offer benefit to the individual [35].

Our findings also suggest that distraction detachment rumination does not mediate the relationship between ICU stressors and ill health. Studies have found that psychological detachment or mentally distancing oneself from work has positive impacts on mood and low fatigue; however, high time pressure and high workload can make it difficult to psychologically detach from work [36]. Psychological detachment acts as a potential buffer of the negative impacts of job stressors on strain reaction [37], while other studies have found that psychological detachment does not mediate the relationship between job demands and cognitive failures [38]. In the current study, the impact of individual ICU stressors on rumination styles, and the impact of work–home conflict or family–work conflict (family or home responsibilities interfering with work) on rumination styles, was not investigated.

There are some limitations that need to be considered. Data collection was restricted to four intensive care units in the South of England and an online survey to doctors, which limits the generalizability of the findings. It is possible that the self-selecting nature of the sample may have resulted in those experiencing high levels of stress and burnout not participating in the study. Even though the study took into account the different dimensions of rumination, the frequency and duration of the ruminating thought was not explored. Further to this, relatively low reliability indicators for the WRRQ were obtained in this sample, particularly for the problem-solving pondering subscale. Although the three subscales of this questionnaire have shown good reliability and clear factor loadings in the previous studies, the scale is relatively new and has not yet been widely used, particularly in more diverse samples. Further research is needed to determine whether modifications of this questionnaire are needed, as the low inter-item reliability of this subscale may have contributed to null mediation findings for this subscale in the current study. Finally, our study included a specific sample of healthcare professionals working in ICU; it would be interesting to replicate our findings in other healthcare professionals facing a high workload and high emotional demands.

## Conclusions

This study demonstrates the potential value for screening healthcare practitioners within intensive care in order to provide targeted interventions. Affective rumination can act as a precursor to developing psychological problems, such as depression and anxiety and long-term health consequences, including cardiovascular disease and other chronic conditions [39]. Screening and identifying those with an affective rumination style and those working in excess of 40 h per week may protect healthcare practitioners from burnout, depression and psychiatric morbidity.

Intervention programmes to reduce burnout found that person-directed interventions, such as cognitive behavioural therapy (CBT) and relaxation exercises, led to a significant reduction in burnout, in comparison with organisational-directed interventions [40]. These person-directed interventions can effectively reduce negative rumination styles, such as affective rumination, and encourage recovery from work [41]. There may be a potential to further develop mindfulness meditation for ICU professionals with affective rumination styles to prevent stress, which could enable them to pay attention in the present moment, rather than react later with negative feeling [17]. The quality of care for ICU patients and their relatives may be compromised through long-term absenteeism or skill drain if healthcare professionals leave their jobs prematurely to preserve their health, ultimately leading to economic burdens [17].
